# Comparisons of Feed Bunk Nutrient Consistency, Milk Production and Cow Behavior Between Herds Using Automated Milking Systems With or Without Automated Feeding Robots

**DOI:** 10.3390/ani15081103

**Published:** 2025-04-11

**Authors:** Kevin Kamau, Benjamin Thorpe, Katie E. Meier, Marcia I. Endres, Isaac J. Salfer

**Affiliations:** 1 Department of Animal Science, University of Minnesota, St. Paul, MN 55108, USA; kamau022@umn.edu (K.K.); miendres@umn.edu (M.I.E.); 2 Lely North America, Pella, IA 50219, USA; bthorpe@lely.com (B.T.); kmeier@lely.com (K.E.M.)

**Keywords:** precision feeding, robotic feeding system, dairy technology, automated milking system

## Abstract

Automated feeding robots (AFR) are increasingly being used on North American dairy farms to reduce dependency on human labor for feeding. These systems mix, deliver, and push up feed to cows at any frequency or interval desired, allowing for more frequent feed delivery than conventional feeding systems (CFS). This observational study investigated differences in ration consistency, milk components, milk fatty acid profile, and cow behavior between herds using AFR and those using CFS. Sixteen commercial dairies with automated milking systems (AMS) in the upper Midwest United States were paired based on herd size and location into eight blocks each consisting of one CFS and one AFR herd. Feed bunk samples were collected at four equally spaced time points for 3 consecutive d and analyzed for coefficient of variation (CV) of nutrient composition and particle size distribution. Bulk tank milk samples were collected 1 ×/d for 3 d and analyzed for fat, protein, milk urea nitrogen (MUN), lactose, and milk fatty acid (FA) profile. Daily AMS visit intervals, milk yield and composition, and rumination time data were collected from AMS software. A linear mixed model tested fixed effects of feeding system, block, and the random effect of day nested within block. The CV of feed bunk DM, ADF, NDF, and lignin was lower in AFR. Bulk tank milk fat, protein, and MUN were not different between AFR or CFS. AFR had a greater proportion of de novo synthesized FA, but no difference in preformed or mixed FA. Herds with AFR had a shorter AMS visit interval with more AMS refusals per day than CFS. Results imply that AFR may be associated with lower daily variation in fiber concentration at the feed bunk, increased mammary de novo fatty acid synthesis, and increased frequency of cow visits to the AMS compared to conventional PMR feeding.

## Simple Summary

Automated feeding robots are receiving increased interest as a way to reduce labor costs and control timing and frequency of feed delivery. However, research on these systems is limited. This observational study compared daily feed bunk nutrient composition consistency, milk components, fatty acid profile, and cow behavior between herds using automated feeding robots and conventional feed mixers. Results suggest that herds using automated feeding robots had lower daily variation in feed bunk dry matter and fiber, greater proportions of milk de novo fatty acids, and more frequent visits to the automated milking system compared with herds using conventional feeding systems.

## 1. Introduction

Automated precision technologies have received heavy adoption by dairy producers within the past several years, both in the United States and globally [1]. These technologies include automated feeding systems (AFS) and automated milking systems (AMS) which allow producers to optimize labor and collect additional herd performance metrics compared to conventional feeding (CFS) and milking systems [2]. Automated feeding systems were commercially introduced in Europe in 2004 [3]. Adoption has since expanded across Europe and North America, primarily due to decreased availability and increased costs of labor [1]. While early AFS used low-volume rail-guided mixer wagons, self-propelled mixer feeder-wagons, referring to automated feeding robots (AFR), have increased in popularity in recent years [4]. Within these systems, some or all feed ingredients are automatically added to these mixer-feeder wagons using a conveyor belt, elevator, or crane assembly with a feed grabber. These systems are designed to precisely control the addition of ingredients and reduce human error in weight measurements.

In addition to reduced labor costs, AFR provide the additional advantage of allowing increased frequency of feed delivery beyond the 1 or 2 ×/d that is standard for most dairy farms. Bisaglia et al. (2012) found that in 22 European herds (10 CFS and 12 rail-guided AFS herds), AFS delivered an average of 7.8 ×/d, with a range of 5 to 11 ×/d [4]. Increasing feeding frequency provides several benefits to dairy farms, including increased feed intake, reduced sorting, improved feed efficiency, and improved milk and milk fat production [5,6]. Delivery of fresh feed stimulates feed intake in dairy cows, and increased feeding frequency has been associated with a greater number of meals per day and more stable rumen pH [7]. Reduction in daily variations in rumen pH, specifically a greater minimum rumen pH, are associated with greater milk de novo fatty acid synthesis due to lower production of alternative biohydrogenation intermediates including *trans*-10 *cis*-12 conjugated linoleic acid (CLA), which inhibits lipogenesis in the mammary gland [8]. Furthermore, increasing frequency of feed delivery is associated with reduced feed sorting on commercial dairy farms [9]. The increased feeding frequency afforded by AFS may improve feed efficiency and milk fat concentration and improve feed bunk total mixed ration (TMR) consistency compared to conventional feeding systems.

To date, limited research has examined the impacts of using automated mixing and feeding robots on feeding consistency, cow performance, or cow behavior. Belle et al. (2012) compared AMS visits between herds using AFS (11 total: 6 self-propelled and 5 rail-guided systems) and those using CFS (9 total) [10]. They observed that herds using AFS had more frequent visits to the AMS in early morning, but no differences in the total number of milkings, milking failures, or refusals. Other reports from Europe have considered impacts of AFS on labor costs and energy consumption, but not on feed consistency or milking performance [11,12,13]. Therefore, the objective of this observational study was to compare herds using AFR and CFS for consistency of feed bunk nutrient concentration and particle size distribution, milk fat percentage, milk FA profile, rumination time, and AMS visit frequency. We hypothesize that herds using AFR have more consistent feed bunk nutrient composition compared to CFS herds and greater milk fat and de novo FA concentrations, indicating more consistent rumen fermentation across the day.

## 2. Materials and Methods

### 2.1. Farms and Experimental Design

Sixteen dairy farms with AMS in the upper Midwest United States (Minnesota, Iowa, and Wisconsin) were assigned to one of 8 blocks based on geographical location and herd size (Table 1). Herds were paired by feeding system, with one herd per block utilizing a manually operated TMR mixer to deliver partial mixed ration (PMR) 1 or 2 × daily (CFS; n = 8) and one herd per block utilizing an automated mixing and feeding robot (AFR; Vector, Lely Industries N.V., Maassluis, The Netherlands [patent no. NL2012856B1]; n = 8). While sample size was determined based on availability of herds, we were able to achieve at least an 80% power of observing *p* < 0.05 for a difference of 2.5 g/100 g FA in de novo fatty acid concentrations between AFR and CFS herds based on a standard deviation of 1.22 g/100 g FA [14]. All farms used freestall housing and AMS (Astronaut, Lely Industries N.V.) with a free-flow cow traffic design. Herds were required to have utilized AMS for at least 6 months prior to the start of the experiment, and AFR herds were required to have used AFR for at least 1 year. Feed was regularly pushed up in the feed bunk for all herds, with CFS herds utilizing automated feed pushers (Lely Juno, Lely Industries N.V.), and AFR herds utilizing AFR to push up feed. All herds fed a single ration to all lactating cows and had either 1 or 2 pens with 1 to 2 AMS per pen (58.7 ± 8.4 cows/AMS; mean ± standard deviation). No herds had a designated fresh cow pen, and all lactating cow pens were sampled across all herds. The average herd size was 138 lactating cows, and all herds had Holstein as the predominant breed. Researchers ensured that no major nutritional changes (e.g., diet reformulation or change in forage source) were made on any herds within 2 weeks prior to sample collection. Additional demographic information of the herds within the study is provided in Table 1. Researchers were not allowed access to the specific number of feedings per day for AFR herds, but AFR herds fed an average of 10.7 ×/d (range: 4.5 to 17.8 ×/d; median: 11.0 ×/d). On-farm data were collected by the same researcher for all herds, and all herds were sampled within the same 6-month time period between August 2021 and February 2022.

### 2.2. Feed Sampling and Analysis

Samples were collected from feed bunks of the 2 farms within each block 4 ×/d at equally spaced time points (approximately 0500, 1100, 1700, and 2300) for 3 consecutive days. Due to travel time between farms, samples from the 2 herds within each block were collected within 1 h of each other. At each sampling time, samples were manually collected into a 5-gallon bucket from sites at 12 evenly spaced intervals across the length of the feed bunk(s) in a consistent zigzag pattern, with ends of the bunks avoided. Samples were mixed and subsampled using the quartering technique. Briefly, samples were thoroughly mixed for 3 min and divided into 4 equal subsamples. Then, 2 subsamples allocated diagonally were re-homogenized and saved. One subsample was used for particle size separation, performed immediately after collection using a 4-compartment Penn State Particle Separator (PSPS) with 19-, 8-, and 4-mm sieves. The other subsample was frozen at −20 °C immediately after collection for analysis of nutrient composition. Association of Official Analytical Chemists (AOAC) International wet chemistry methods [15] were used to determine concentrations of dry matter (DM; method 930.15) crude protein (CP; 990.03), soluble protein (SP; [16]) neutral detergent insoluble nitrogen (NDIN; 2002.04 followed by 990.13), acid detergent insoluble nitrogen (ADIN; 973.18 followed by 990.03), amylase-treated neutral detergent fiber (NDF; 2002.04), acid detergent fiber (ADF; 973.18), lignin (973.18), starch (920.40), ethanol-soluble carbohydrate (ESC; 982.14), total FA (996.06), and ash (942.05) at a commercial laboratory (Dairyland Laboratories, St. Cloud, MN, USA). The same researcher performed all sampling, subsampling, and particle size separation, and care was taken to ensure that samples were consistent and representative of all feed particles within the bunk. Daily coefficients of variation (CV) of feed bunk nutrient composition and the proportion of PMR in each compartment of the PSPS were determined for each of the 3 days by determining the standard deviation of the 4 time points within a day (0500, 1100, 1700, and 2300) and dividing by the mean of those 4 samples for each herd on each day of sampling.

### 2.3. Bulk Tank Sampling and Analysis

Bulk tank milk samples were collected on the same days as feed bunk sampling, 1 ×/d for 3 consecutive days immediately prior to milk pickup. For all herds, milk was either picked up once per day, or a separate bulk tank was used for each day of milking, so each milk sample represented 1 day of milk for the entire lactating herd. Samples were collected after turning on the bulk tank mixer for 5 min using a stainless-steel ladle to collect a sample from 1 foot below the surface of the milk and pouring the milk into 50 mL collection vials for further analysis. One aliquot was refrigerated for a maximum of 4 d before analysis of fat, protein, lactose MUN, and SCC concentration using Fourier-Transform Infrared Spectroscopy (FTIR; Fossomatic 400 Milko-Scan and 400 Fossomatic, Foss Electric; Minnesota DHIA Laboratories, Sauk Centre, MN, USA), while the other was immediately frozen and stored for analysis of individual fatty acid concentrations. Milk was thawed at room temperature, and FA were extracted using hexane-isopropanol followed by transmethylation in the presence of sodium methoxide. Resulting fatty acid methyl esters (FAME) were quantified using gas chromatography with flame ionization detection. Samples were injected into a 100 m fused silica capillary column (SP-2560, 100 m × 0.25 mm i.d. with 0.2 μm film thickness; Supelco Inc., Bellefonte, PA, USA). Hydrogen was used as the carrier gas (flow: 1 mL/min). Initial oven temperature was 80 °C, which was increased 2 °C/min to 190 °C and held for 15 min. Inlet and detector temperatures were 250 °C with a 100:1 split gas ratio. Detector hydrogen flow was held at 25 mL/min, airflow was 400 mL/min, and nitrogen plus carrier gas flow was 40 mL/min. Peaks were identified using the following FAME standards: GLC461, GLC 780 (Nu-Check Prep Inc., Elysian, MN, USA), and pure *trans*-10, *cis*-12 CLA, and *cis*-9 *trans*-11 CLA (Matreya LLC., State College, PA, USA). An equal weight reference standard (GLC 74; Nu-Chek Prep Inc.) was used to determine correction factors for individual fatty acids.

### 2.4. AMS Milk Production and Composition, Milking Interval, Refusals, AMS Pellet Intake, and Rumination

Daily milk production and composition estimates, AMS visit behavior variables and robot feed allocation were collected from AMS software (Time for Cows [T4C] 3.11, Lely Industries, Maassluis, The Netherlands) beginning 1 week prior to and ending 1 week following collection of bulk tank and feed bunk PMR samples for each herd. Real-time rumination was recorded by wireless accelerometer neck collars (SCR Heatime HR, Allflex Monitoring, Madison, WI, USA) previously validated by Kappes et al. [17], and total rumination time/cow/d was calculated using T4C 3.11 software. Indications of milk fat and protein concentrations were determined by near-infrared spectroscopy in-line sensors within the AMS during each milking (Astronaut, Lely Industries) [18]. Milk was weighed by each individual milking and summed to determine total milk per cow produced within a day, while a weighted average of component concentrations was determined within each day. In-line sensors were calibrated against bulk tank values at least weekly. Estimated fat and protein yields were calculated by multiplying milk yields by indications of fat and protein concentrations. Automated milking system visit parameters included visit interval (average time h between each visit to AMS), AMS milking interval (average time h between successful visits where milking unit was attached), and AMS refused visits (number of visits to the AMS/cow/day where no milking was allowed because of milking permission settings). Individual cow data were averaged by day within each herd prior to statistical analysis.

### 2.5. Statistical Analysis

All data were analyzed using linear mixed effects models in JMP Pro 16 (SAS Institute, Cary, NC, USA). The main effects and interaction of sampling time and feed bunk nutrient composition and particle size distribution was analyzed using the following model:

*y_ijklm_* = *µ* + *S_i_* + *T_j_* + *(S* × *T)_ij_* + *B_k_* + *D_l(k)_* + *H_m(k)_* + *ε_ijklm_* (1)

where *y_ijklm_* is the response variable of interest, *μ* is the overall mean, *S_i_* is the fixed effect of feeding system (CFS vs. AFR), *T_j_* is fixed effect of time of day (0500, 1100, 1700, and 2300), *(S × T)_ij_* is the fixed effect of the interaction between feeding system and time of day, *B_k_* is the fixed effect of block, *D_l(k)_* is the random effect of day nested within block, *H_m(k)_* is the random effect of herd nested within block, and *ε_ijklm_* is the residual error. Post hoc multiple comparisons for the treatment by time interaction were performed using Tukey’s honest significant difference (HSD) test. Coefficient of variation of nutrient composition and particle size distribution, milk yield and component concentrations, feed allocation, AMS visits and refusals, and rumination were analyzed using following model:

*y_ijkl_* = *µ* + *S_i_* + *B_j_* + *D_k(j)_* + *H_l(j)_* + *ε_ijkl_* (2)

where *y_ijkl_* is the response variable of interest, *μ* is the overall mean, *S_i_* is the fixed effect of feeding system, *B_k_* is the fixed effect of block, *D_l(k)_* is the random effect of day nested within block, *H_m(k)_* is the random effect of herd nested within block, and *ε_ijkl_* is the residual error. Average days in milk (DIM) and lactation number were initially included as covariates but were removed due to a lack of statistical significance (*p* > 0.10). The autoregressive [AR(1)], compound symmetry (CS), and variance components (VC) covariance structures were tested, and AR(1) was selected based on Bayesian information criterion (BIC). Denominator degrees of freedom were adjusted using the Kenward–Roger method. Significance was declared at *p* < 0.05 with tendencies declared at 0.05 < *p* < 0.10. Normality of residuals was determined by histogram and normal Q-Q plot.

## 3. Results and Discussion

### 3.1. Consistency of Feed Bunk Nutrient Composition

Average nutrient concentrations of the PMR did not differ between CFS and AFR for any nutrient measured (*p* > 0.12; Table 2). The concentration of feed particles on the lower sieve (8 to 4 mm) of the PSPS tended to be greater in AFR than CFS (*p* < 0.06), but concentration of feed particles on the other sieves did not differ by feeding system. This increase in small particles may be indicative of a greater particle size reduction in the AFR mixers compared to conventional TMR mixers. Herds with AFR had a 33% lower coefficient of variation in DM (*p* = 0.02) and a 15% lower variation in ADF concentration compared to CFS (5.20 vs. 7.08; *p* = 0.04; Table 3). Similarly, variation in NDF concentration tended to be lower in herds using AFR compared to those with CFS (4.53 vs. 6.10; *p* = 0.09). There were no differences in variation of lignin, CP, SP, ADIN, NDIN, starch, ethanol-soluble carbohydrates, total FA, or ash between feeding systems (*p* > 0.15). Furthermore, no differences in distribution on any of the four compartments of the PSPS were observed.

Previous reports have shown that feed sorting can be reduced by more frequent feed delivery. DeVries et al. (2005) observed that NDF concentration of feed at the feed bunk increased over the day to a greater extent in herds fed 1 ×/d compared to those being fed 2 × or 4 ×/d, indicating that cows were sorting for lower NDF feeds [19]. Similarly, Hart et al. (2014) observed a reduction in sorting of small particles when cows were fed 3 ×/d compared to 1 × and 2 ×/d feeding [7]. Furthermore, Endres and Espejo, (2010) found that across 50 freestall herds in Minnesota, feeding 2 ×/d or more was associated with greater consistency of NDF concentration at the feed bunk compared to feeding 1 ×/d [9].

While feeding frequency was not directly controlled within our study, herds using AFR fed an average of 10.7 ×/d (range: 4.5–17.8 ×/d; median: 11.0 ×/d) compared to 1 to 2 ×/d in CFS. The opportunity to deliver fresh feed more frequently may have reduced sorting in AFR herds, leading to more consistent nutrient composition at the feed bunk. Over time, some feed moisture volatilizes, reducing the DM concentration of forages [20]. Potential increased frequency of feed delivery may have reduced moisture volatilization in AFR herds. Automation of feed mixing may have also improved ration consistency over time. Despite the differences in DM, NDF, and ADF concentrations, no differences were observed in CV of particle size distribution between feeding systems (*p* > 0.20).

We designed our experiment to collect PMR samples based on the time of day rather than time relative to feeding. This approach was necessary because the AFR herds in our study did not have fixed feeding times; instead, they were fed based on estimated feed levels measured using a laser sensor. Consequently, there was no definitive feeding time in the AFR herds, and sampling based on the CFS herds’ feeding schedule could have biased the sorting data in favor of the AFR. To address this, we chose to sample at four equally spaced time points throughout the day. While this prevented us from measuring feed sorting immediately after feed delivery, it allowed us to assess variations in nutrient composition and particle size within the feed bunk throughout the day.

### 3.2. Bulk Tank Milk Composition

Milk composition was determined during the same days as TMR nutrient composition was determined (Table 4). No differences in milk fat, protein, lactose, urea N, or somatic cell count concentrations were observed between CFS and AFS (*p* > 0.20). Our results concur with Da Borso et al. (2017), who observed no differences in milk production between herds with AFR and conventional feeding systems [12]. Future research focused on studying the impacts of AFR on production should be performed in more controlled environments without other potential confounding effects across herds.

### 3.3. Bulk Tank Milk Fatty Acid Profile

Milk FA can originate from uptake of preformed FA from plasma (>16 carbons), de novo synthesis in the mammary gland (<16 carbons), or mixed origin (16 carbons; [21]). Herds with AFR had a 1 g/100 g greater proportion of de novo synthesized FA (Σ < 16C FA) in bulk tank milk compared to herds with CFS (*p* = 0.03; Table 5). Specifically, concentrations of C10:0, C12:0, and C:14:0 were 7.0%, 7.4%, and 3.9% greater in AFR compared to CFS (*p* = 0.01). Alternately, proportions of total preformed (Σ > C16; *p* = 0.32), and mixed (Σ C16; *p* = 0.41) fatty acids did not differ between CFS and AFR. Furthermore, none of the individual preformed or C16 FA differed by feeding system (*p* > 0.10).

De novo synthesized fatty acids can be increased by availability of fatty acid precursors—mainly acetic acid, or by a reduction in biohydrogenation-induced milk fat depression caused by *trans*-10 *cis*-12 conjugated linoleic acid (*t*10, *c*12 CLA) [8]. Increased time with rumen pH below 5.8 is associated with greater production of t10 c12 CLA, resulting in lower de novo fatty acid synthesis [22]. Again, while feeding frequency was not directly controlled within our study, the average increase in feeding frequency allowed by AFR may have contributed to increased de novo fatty acids. Rottman et al. (2014) observed increased amounts of de novo synthesized fatty acids in cows fed 4 ×/d compared to those fed 1 ×/d [23]. More recently, Castro et al. (2022) observed in 124 Canadian dairy herds that feeding more than 2 ×/d was associated with greater de novo and de novo plus mixed FA concentrations compared to feeding 1 × or 2 ×/d [24]. Alternately, Woolpert et al. (2016) observed no association between feeding frequency based on odds-ratio of high versus low bulk tank de novo fatty acid concentrations [14]. However, herds within that experiment fed cows a maximum of 2 ×/d. Results of our study indicate that the additional feeding frequency allowed by AFR may increase de novo fatty acid synthesis. However, within our experiment, differences in total milk fat concentrations were not observed.

Assessing total concentrations of *trans*-10 C18:1 and trans-11 C18:1 provides insights into the role of ruminal biohydrogenation on de novo fatty acid synthesis. During normal rumen biohydrogenation, the majority of C18:1 is formed as trans-11 C18:1, but in circumstances of low rumen pH, a greater proportion of *trans*-10 C18:1 is produced via alternate biohydrogenation pathways. Therefore, elevated *trans*-10 C18:1 concentrations are associated with biohydrogenation-induced milk fat depression. Despite the difference in de novo fatty acid synthesis, no differences in proportions of *trans*-10 C18:1 (*p* = 0.60), or trans-11 C18:1 (*p* = 0.19) were observed between herds with CFS and those with AFR. Despite a lack of difference in *trans*-10 C18:1 concentration, we expect that the increased de novo fatty acid concentration in AFR herds within our study was due to stabilization of feed intake and reduced biohydrogenation-induced milk fat depression.

### 3.4. AMS Milk Production and Composition, Milking Interval, Refusals, Pellet Intake, and Rumination

Milk yield and estimated milk fat and protein yields did not differ between CFS and AFR herds (*p* > 0.68). As observed with bulk tank milk sampling, there was no difference in milk fat or protein concentration based on feeding system (*p* > 0.18; Table 6). Notably, the fat indications measured using the in-line sensors were 0.19 percentage points higher than the concentrations measured from bulk tank samples using FTIR. This remained true even when AMS fat indications from only the 3 days of bulk tank sample collection were analyzed, with those three days having an average fat indication of 4.01 for CFS and 4.02 for AFR. In contrast, milk protein indications from the 3 sampling days were identical to measured bulk tank concentrations (3.12 for CFS, 3.14 for AFR). Fadul-Pacheco et al. (2018) compared values for milk fat percentage from in-line sensors within an AMS to laboratory FTIR values and found a large variance in sensor accuracy among herds, with one herd having a 0.22 percentage point underestimation of laboratory values by the AMS sensors, and another having a 0.14 percentage point overestimation [18]. Similar to our results, the discrepancy was much smaller for milk protein percentage, ranging from just a 0.02 percentage point underestimation to a 0.07 percentage point overestimation. It has been reported that free fatty acids are increased in AMS, likely due to increased milking frequency [25]. The specific lack of precision in the measurement of milk fat concentration may be due to these changes in the physical properties of milk affecting the accuracy of measurement.

On average, the visit interval (time between consecutive voluntary visits to the AMS) of cows in AFR herds was 19.2 min shorter than those in CFS herds (*p* < 0.001; Table 6). However, AMS refused visits were 43% greater in AFR herds (1.79/cow/d) than CFS herds (1.25/cow/d; *p* < 0.001), resulting in no difference in milking interval between feeding systems (*p* = 0.91). Permission settings imposed by the farm manager dictate the minimum interval between milkings of an individual cow, and cows visiting earlier than their allowable interval are ‘refused’ or dismissed from the AMS without being milked [26]. The increased number of cow visits suggests that frequent feeding or the presence of the AFR stimulated cow activity within the freestall barn. Anecdotal evidence from several of the individual farm managers noted increased cow activity after implementation of the AFR, but no quantification of this change was made. Delivery of fresh feed is known to stimulate cow activity as cows travel to the feed bunk [27]. However, Oostra et al. (2005) observed no change in AMS visits or refusals when cows were switched from 2 ×/d feeding to 6 ×/d feeding using a conventional TMR mixer [28]. Moreover, Belle et al. (2012) observed no differences in refused visits between AFR or CFS [10]. Future research quantifying cow activity within herds using AFR would provide valuable insights into the impacts of automated feeding on cow activity within an AMS system.

We observed a 12.1% reduction in feed allocation/cow/d within the AMS in herds using AFR compared to those using CFS (*p* < 0.001). Intake within the AMS is primarily determined by producer-created feed tables that designate feed allocation based on a variety of management factors, including forage availability, amount of purchased feed, intensity of feed management, and preferences regarding numbers of “fetch cows” [29]. The decrease in feed allocation was likely associated with shorter AMS visit interval and greater refusals in AFR herds, with more frequent cow visits necessitating less feed needed to incentivize cows to enter the milking robot.

Rumination time is determined by the NDF concentration and mean particle size of the diet and can be influenced by feeding time and feeding frequency [17]. We observed no difference in daily rumination time between CFS and AFR herds (484 min/d in CFS vs. 515 min/d in AFR; SEM = 17.5; *p* = 0.27). Previous reports suggest that increasing frequency of eating decreases rumination time. Phillips and Rind (2001) detected increased rumination time of cows when feed delivery was decreased from once per day to once every two days [30]. Our results suggested that the increased feed delivery in AFR systems had no positive or negative effect on time spent ruminating.

## 4. Conclusions

Within the conditions of our study, herds using AFR had lower daily variation in feed bunk DM and fiber concentration as well an increased relative proportion of de novo synthesized fatty acids within milk. Furthermore, the frequency of visits to the AMS appeared to increase in AFR herds, perhaps due to greater activity due to stimulation of feed intake by frequent feed delivery. Our study was limited by the small number of herds utilizing AFR at the time of data collection. While we paired herds of similar size and location to try to control for inherent variation, several management factors including specific ration composition, feeding time, feed push-ups, and cow fetching frequency still confounded results. Moreover, the inability to control or account for the specific number of times that the AFR delivered feed during each day limits our interpretation. Future research in controlled settings investigating cow-level milk yields and component concentrations, behavior, and feed intake will provide additional understanding about the impacts of automated feeding robots on cow performance. However, our study provides compelling evidence to suggest that utilization of AFR may reduce variation in nutrient intake across the day and increase de novo fatty acid synthesis.

## Figures and Tables

**Table 1:** Demographic information of herds used within the experiment.

Block	Dates Visited	Location	# of AMS ^1^		# of Cows in Tank		Cows/AMS		Ave DIM ^2^		Average Parity		Bunk Space, cm/cow		Times Fed/d ^3^
			CFS	AFR	CFS	AFR	CFS	AFR	CFS	AFR	CFS	AFR	CFS	AFR	CFS
1	9 August 2021 to 12 August 2021	NE IA	2	3	111	188	56	63	157	190	2.6	2.6	42.7	36.1	2
2	28 August 2021 to 30 August 2021	Central WI	4	2	207	116	52	52	204	181	1.8	2.1	70.6	59.2	2
3	28 August 2021 to 30 August 2021	Central WI	2	1	113	65	57	44	189	131	2.2	2.5	76.4	56.6	2
4	2 October 2021 to 4 October 2021	Central MN	2	3	134	181	67	60	164	193	2.0	2.2	44.4	45.5	2
4	16 October 2021 to 19 October 2021	SE MN	2	2	125	125	63	63	151	194	2.1	2.2	52.8	81.0	1
6	24 November 2021 to 26 November 2021	NW MN	3	3	132	177	44	59	240	208	2.1	2.3	45.0	39.9	2
7	27 November 2021 to 29 November 2021	NW MN	2	3	154	170	77	57	196	205	2.5	2.8	56.6	48.3	2
8	31 January 2022 to 3 February 2022	Central MN	2	3	200	116	67	58	188	195	2.5	2.6	64.0	44.7	1
Average			2.5	2.4	147	142	60.4	57.0	186	187	2.2	2.4	56.6	51.3	1.75
Standard Deviation			0.74	0.75	37.4	43.4	10.3	6.3	29.0	24.2	0.28	0.24	12.7	14.2	0.46

^1^ Indicates number of automated milking systems for herds using either conventional PMR feeding using a manually driven TMR mixer delivered to cows 1 or 2 ×/d (CFS; n = 8) or an automated mixer-feeder robot that automatically mixed and delivered feed 4 to 18 ×/d (AFR; n = 8). ^2^ Average days in milk of cows of cows used for data collection. ^3^ Herds using AFR fed an average of 10.7 ×/d (range: 4.5 to 17.8 ×/d; median: 11.0 ×/d).

**Table 2:** Comparisons of feed bunk nutrient composition and particle size distribution across the day between herds with conventional feeding systems or automated feeding robots.

	Feeding System ^1^								SEM ^2^	*p*-Value ^3^		
	CFS				AFR							
	0500 h	1100 h	1700 h	2300 h	0500 h	1100 h	1700 h	2300 h		System	Time	S × T
Feed Bunk Nutrient Composition ^4^												
Dry Matter, %	46.9	47.6	47.8	47.6	45.8	46.1	45.9	45.7	1.44	0.44	0.33	0.57
Crude Protein, % of DM	15.7	15.9	15.8	15.7	15.7	15.7	15.5	15.5	0.34	0.68	0.51	0.64
Soluble Protein, % of DM	6.38	6.36	6.57	6.51	6.27	6.18	6.15	6.21	0.37	0.64	0.54	0.12
Acid Detergent Insoluble Nitrogen, % of DM	1.40	1.42	1.37	1.38	1.42	1.43	1.42	1.43	0.029	0.42	0.13	0.35
Neutral Detergent Insoluble Nitrogen, % of DM	2.57	2.65	2.44	2.46	2.53	2.56	2.47	2.54	0.17	0.98	0.01	0.24
Acid Detergent Fiber, % of DM	22.6	23.1	22.0	22.5	23.1	23.3	23.3	23.6	0.74	0.54	0.54	0.52
Neutral Detergent Fiber, % of DM	31.3	31.8	30.4	31.2	32.6	32.8	33.1	33.5	1.34	0.35	0.36	0.17
Lignin, % of DM	4.09	4.23	4.02	4.11	4.21	4.25	4.27	4.28	0.15	0.48	0.54	0.42
Starch, % of DM	25.4	24.9	25.4	25.0	24.6	24.0	24.0	24.2	1.10	0.61	0.73	0.95
Ethanol-Soluble Carbohydrate, % of DM	4.10 ^xy^	4.01 ^y^	4.49 ^x^	4.18 ^xy^	4.92 ^x^	4.75 ^x^	4.81 ^x^	4.55 ^x^	0.38	0.30	0.05	0.08
Total Fatty Acids, % of DM	2.90	2.87	2.90	2.87	2.78	2.76	2.77	2.76	0.10	0.41	0.95	0.99
Ash, % of DM	8.35	8.33	8.39	8.30	7.79	7.73	7.72	7.72	0.24	0.12	0.78	0.80
Feed Bunk Particle Size Distribution ^5^												
Upper Sieve (>19 mm), %	21.4	21.8	20.4	21.5	23.1	22.4	22.4	24.5	4.25	0.76	0.58	0.77
Middle Sieve (19 to 8 mm), %	36.5	36.6	37.1	37.4	35.3	36.0	35.5	35.0	3.50	0.78	0.96	0.75
Lower Sieve (8 to 4 mm), %	13.5 ^y^	13.6 ^y^	13.5 ^y^	12.8 ^y^	16.2 ^x^	16.4 ^x^	18.2 ^x^	15.9 ^x^	1.27	0.06	0.31	0.59
Bottom Pan (<4 mm), %	28.6	28.0	28.9	28.3	25.3	25.2	23.9	24.6	1.94	0.19	0.91	0.52

^x–y^ Means with differing superscripts within rows tend to differ at 0.05 < *p* < 0.10. ^1^ Feeding systems included manually driven TMR mixers delivering PMR to cows 1 or 2 ×/d (CFS; n = 8) or a self-propelled automated mixer-feeder robot delivering PMR 4.5 to 17.8 ×/d (AFR; Vector, Lely Industries; n = 8). ^2^ SEM: Standard error of the mean (n = 8/group) ^3^ *p*-values of the main effects of feeding system (System), time of day (Time) and their interaction (S × T). Significance was declared at *p* < 0.05 with tendencies declared at 0.05 < *p* < 0.10. ^4^ Samples were collected from the feed bunk 4 ×/d at 6 h intervals (0500, 1100, 1700, 2300) over 3 consecutive days. Nutrient composition was analyzed using appropriate wet chemistry methods according to AOAC Int’l (Dairyland Laboratories, St. Cloud, MN, USA). ^5^ Percentage of particles within each compartment of a 4-compartment Penn State Particle Size Separator (PSPS).

**Table 3:** Comparisons of daily variation in feed bunk nutrient composition and particle size distribution between herds with conventional feeding systems or automated feeding robots.

	Feeding System ^1^		SEM ^2^	*p*-Value ^3^
Item	CFS	AFR		
Daily Variation in Feed Bunk Nutrient Composition ^4^				
CV of Dry Matter, %	3.08	2.06	0.24	0.02
CV of Crude Protein, %	2.50	3.23	0.32	0.15
CV of Soluble Protein, %	5.27	3.40	0.94	0.22
CV of Acid Detergent Insoluble Nitrogen, %	4.53	3.08	0.72	0.20
CV of Neutral Detergent Insoluble Nitrogen, %	7.33	6.03	0.86	0.26
CV of Acid Detergent Fiber, %	7.08	5.20	0.53	0.04
CV of Neutral Detergent Fiber, %	6.01	4.53	0.60	0.09
CV of Lignin, %	7.40	6.26	5.19	0.40
CV of Ethanol-Soluble Carbohydrate, %	13.0	8.42	3.08	0.32
CV of Starch, %	6.44	5.38	0.65	0.32
CV of Total Fatty Acids, %	7.34	8.75	0.74	0.21
CV of Ash, %	2.92	3.30	0.50	0.61
Daily Variation in Feed Bunk Particle Size Distribution ^5^				
CV of Upper Sieve (>19 mm), %	23.3	26.7	3.02	0.41
CV of Middle Sieve (19 to 8 mm), %	8.84	10.3	1.78	0.57
CV of Lower Sieve (8.0 to 4 mm), %	6.80	12.1	2.74	0.20
CV of Bottom Pan (<4 mm), %	10.7	13.4	2.15	0.33

^1^ Feeding systems included manually driven TMR mixers delivering PMR to cows 1 or 2 ×/d (CFS) or a self-propelled automated mixer-feeder robot delivering PMR 4.5 to 17.8 ×/d (AFR; Vector, Lely Industries). ^2^ SEM: Standard error of the mean (n = 8/group). ^3^ *p*-value of the main effect of feeding system. Significance was declared at *p* < 0.05 and tendencies at 0.05 < *p* < 0.10. ^4^ Daily coefficient of variation (CV; standard deviation/mean) of feed bunk nutrient composition determined from 4 samples collected at 6 h intervals (0500, 1100, 1700, 2300), determined each day over 3 consecutive d. Nutrient composition was analyzed using appropriate wet chemistry methods according to AOAC International (Dairyland Laboratories, St. Cloud, MN, USA). ^5^ Daily CV of the proportion of feed on each compartment of a 4-compartment Penn State Particle Size Separator (PSPS) determined from 4 samples collected at 6 h intervals (0500, 1100, 1700, 2300), determined each day over 3 consecutive d.

**Table 4:** Comparison of bulk tank milk composition between herds with conventional feeding systems or automated feeding robots.

	Feeding System ^2^		SEM ^3^	*p*-Value ^4^
Item ^1^	CFS	AFR		
Milk Fat concentration, %	3.86	3.80	0.046	0.47
Milk True Protein concentration, %	3.12	3.12	0.020	0.98
Milk Lactose concentration, %	4.79	4.82	0.015	0.20
Milk Urea Nitrogen, mg/dL	11.4	11.6	0.98	0.90
Somatic Cell Count, 1000 cells/mL	201	240	36.5	0.47

^1^ Bulk tank nutrient composition determined using Fourier-Transform Infrared Spectroscopy (FTIR; Minnesota DHIA Laboratories, Sauk Centre, MN, USA). ^2^ Feeding systems included manually driven TMR mixers delivering PMR to cows 1 or 2 ×/d (CFS) or a self-propelled automated mixer-feeder robot delivering PMR 4.5 to 17.8 ×/d (AFR; Vector, Lely Industries). ^3^ SEM: Standard error of the mean (n = 8/group). ^4^ *p*-value of the main effect of feeding system. Significance was declared at *p* < 0.05 with tendencies declared at 0.05 < *p* < 0.10.

**Table 5:** Comparison of bulk tank milk fatty acid profile between herds with conventional feeding systems or automated feeding robots.

	Feeding System ^2^		SEM ^3^	*p*-Value ^4^
FA, g/100 g of Total FA ^1^	CFS	AFR		
C4:0	4.75	4.77	0.088	0.85
C6:0	2.45	2.52	0.056	0.39
C8:0	1.28	1.34	0.025	0.12
C9:0	0.049	0.050	0.0041	0.92
C10:0	2.84	3.04	0.048	0.01
C10:1, *cis*-9	0.26	0.26	0.0065	0.74
C11:0	0.080	0.085	0.0074	0.63
C12:0	3.24	3.48	0.053	0.01
C13:0, *iso*	0.023	0.022	0.0016	0.67
C13:0, *anteiso*	0.074	0.074	0.0028	0.90
C12:1	0.085	0.087	0.0032	0.62
C13:0	0.12	0.13	0.0084	0.58
C14:0, *iso*	0.090	0.087	0.0072	0.74
C14:0	10.3	10.7	0.095	0.01
C15:0, *iso*	0.16	0.18	0.018	0.59
C15:0, *anteiso*	0.39	0.40	0.013	0.53
C14:1, *cis*-9	0.98	0.95	0.037	0.59
C15:0	1.13	1.12	0.057	0.95
C16:0, *iso*	0.22	0.21	0.015	0.58
C16:0	31.6	30.7	0.81	0.46
C16:1, *cis*-9	1.66	1.51	0.082	0.21
C17:0, *iso*	0.43	0.42	0.0089	0.47
C17:0, *anteiso*	0.38	0.38	0.011	0.98
C17:0	0.41	0.39	0.069	0.78
C17:1, *cis*-10	0.23	0.20	0.010	0.10
C18:0	8.77	7.73	0.88	0.31
C18:1, *trans*-4	0.016	0.015	0.0029	0.74
C18:1, *trans*-5	0.013	0.011	0.0024	0.62
C18:1, *trans*-6 to 8	0.24	0.21	0.052	0.76
C18:1, *trans*-9	0.21	0.22	0.013	0.62
C18:1, *trans*-10	0.048	0.53	0.069	0.60
C18:1, *trans*-11	0.67	0.79	0.067	0.19
C18:1, *trans*-12	0.39	0.30	0.086	0.48
C18:1, *cis*-9	15.9	11.4	3.20	0.35
C18:1, *trans*-15	1.28	2.80	1.98	0.49
C18:1, *cis*-11	0.48	0.38	0.081	0.38
C18:1, *cis*-12	0.40	0.39	0.035	0.99
C18:2, *cis*-9, cis-11	2.43	2.62	0.17	0.44
C18:2, *cis*-9, *trans*-11	0.39	0.42	0.024	0.40
C18:3, *cis*-6, *cis*-9, *cis*-12	0.031	0.0031	0.0023	0.95
C18:3, *cis*-9, *cis*-12, *cis*-15	0.50	0.47	0.037	0.61
C20:0	0.10	0.10	0.0057	0.77
C20:1, *cis*-11	0.041	0.038	0.0050	0.69
C20:2, ω-6	0.017	0.016	0.0040	0.78
C22:0	0.086	0.057	0.023	0.38
C20:3, ω-6	0.040	0.071	0.016	0.33
C20:3, ω-3	0.0026	0.0037	0.0020	0.66
C20:4, ω-6	0.11	0.10	0.028	0.87
C22:1 cis-13	0.041	0.038	0.0055	0.69
C20:5, ω-13	0.043	0.034	0.0032	0.02
C24:0	0.0058	0.012	0.0039	0.25
C24:1, ω-9	0.0065	0.0020	0.0016	0.35
C22:4, ω-6	0.017	0.019	0.0044	0.74
C22:5, ω-3	0.71	0.058	0.0031	0.01
Unknown	4.26	8.27	2.63	0.32
Σ < C16 ^5^	28.3	29.3	0.29	0.03
Σ C16 ^6^	33.5	32.4	0.86	0.41
Σ > C16 ^7^	33.9	29.9	2.63	0.32
Total OCFA ^8^	3.48	3.45	0.092	0.84
Total BCFA ^9^	1.78	1.79	0.048	0.93
Total OBCFA ^10^	5.44	5.25	0.12	0.22

^1^ Bulk tank fatty acid (FA) profile was determined by analyzing fatty acid methyl esters (FAME) using gas chromatography with flame ionization detection. ^2^ Feeding systems included manually driven TMR mixers delivering PMR to cows 1 or 2 ×/d (CFS; n = 8) or a self-propelled automated mixer-feeder robot to deliver feed 4.5 to 17.8 ×/d (AFR; Vector, Lely Industries; n = 8). ^3^ SEM: Standard error of the mean (n = 8/group). ^4^ *p*-value of the main effect of feeding system. Significance was declared at *p* < 0.05 with tendencies declared at 0.05 < *p* < 0.10. ^5^ Sum of total FA less 16 carbons in length (de novo synthesized FA). ^6^ Sum of total FA 16 carbons in length (mixed source FA). ^7^ Sum of total FA greater than 16 carbons in length (preformed source FA). ^8^ OCFA: Odd-chain fatty acids. ^9^ BCFA: Branched-chain fatty acids. ^10^ OBCFA: Odd- and branched-chain fatty acids.

**Table 6:** Comparison of AMS feed intake, milk yield, and milk component indications, rumination, refusals, AMS visit interval, and AMS milking interval collected between herds with conventional feeding systems or automated feeding robots.

	Feeding System ^1^		SEM ^2^	*p*-Value ^3^
Item	CFS	AFR		
Milk yield, kg/d	37.5	37.3	0.25	0.68
Milk fat indication ^4^, %	4.01	4.03	0.010	0.18
Milk protein indication ^4^, %	3.12	3.14	0.020	0.51
Fat yield ^5^, g/d	1503	1499	9.76	0.84
Protein yield ^6^, g/d	1170	1172	7.23	0.90
Feed allocation in AMS, kg/cow/d	5.19	4.56	0.063	<0.001
Rumination ^7^, min/d	484	515	17.5	0.27
AMS Refusals, #/d	1.25	1.79	0.05	<0.001
AMS Visit Interval, h	7.62	7.30	0.27	<0.001
AMS Milking Interval, h	8.64	8.63	0.060	0.91

^1^ Feeding systems included manually driven TMR mixers delivering PMR to cows 1 or 2 ×/d (CFS; n = 8) or a self-propelled automated mixer-feeder robot to deliver feed 4.5 to 17.8 ×/d (AFR; Vector, Lely Industries; n = 8). ^2^ SEM: Standard error of the mean (n = 8/group) ^3^ *p*-value of the main effect of feeding system. Significance was declared at *p* < 0.05 with tendencies declared at 0.05 < *p* < 0.10. ^4^ Indications of milk fat and protein concentrations were determined by in-line sensors within the AMS during each milking (Astronaut, Lely Industries). ^5^ Fat yield = daily milk yield of each cow multiplied by fat indication. ^6^ Protein yield = daily milk yield of each cow multiplied by protein indication. ^7^ Rumination was determined using accelerometer neck collars (SCR Heattime).

## Data Availability

The data that support the findings of this study are available upon request.

## Abbreviations

The following abbreviations are used in this manuscript:

ADF	Acid detergent fiber
ADIN	Acid detergent insoluble nitrogen
AFR	Automated feeding robot
AFS	Automated feeding systems
AMS	Automated milking system
AOAC	Association of Official Analytical Chemists
BCFA	Branched-chain fatty acid
CFS	Conventional feeding system
CLA	Conjugated linoleic acid
CP	Crude protein
ESC	Ethanol soluble carbohydrate
FA	Fatty acid
FAME	Fatty acid methyl esters
FTIR	Fourier-Transform infrared spectroscopy
NDF	Neutral detergent fiber
NDIN	Neutral detergent insoluble nitrogen
OCFA	Odd-chain fatty acid
OBCFA	Odd- and branched-chain fatty acids
PMR	Partial mixed ration
PSPS	Penn State Particle Separator
SP	Soluble protein
*t*10, *c*12 CLA	*trans*-10 *cis*-12 conjugated linoleic acid
TMR	Total mixed ration
